# Synthesis and Antibacterial Activity of Some Heterocyclic Chalcone Analogues Alone and in Combination with Antibiotics

**DOI:** 10.3390/molecules17066684

**Published:** 2012-06-01

**Authors:** Thanh-Dao Tran, Thi-Thao-Nhu Nguyen, Tuong-Ha Do, Thi-Ngoc-Phuong Huynh, Cat-Dong Tran, Khac-Minh Thai

**Affiliations:** 1 Department of Medicinal Chemistry, School of Pharmacy, University of Medicine and Pharmacy at Ho Chi Minh City, 41 Dinh Tien Hoang, Dist. 1, Ho Chi Minh City 70000, Vietnam; Email: thaonhu1986@yahoo.com (T.-T.-N.N.); ngocphuonght@yahoo.com (T.-N.-P.H.); 2 Department of Synthetic/Organic Chemistry, Ton Duc Thang University, Nguyen Huu Tho St., Tan Phong Ward, Dist. 7, Ho Chi Minh City 70000, Vietnam; Email: dtuongha@yahoo.com; 3 Department of Microbiology, School of Pharmacy, University of Medicine and Pharmacy at Ho Chi Minh City, 41 Dinh Tien Hoang, Dist. 1, Ho Chi Minh City 70000, Vietnam; Email: trancdong@gmail.com

**Keywords:** chalcone, heterocyclic chalcone analogues, synthesis, antibacterial, synergitic activity, antibiotics combination, MRSA

## Abstract

A series of simple heterocyclic chalcone analogues have been synthesized by Claisen Schmidt condensation reactions between substituted benzaldehydes and heteroaryl methyl ketones and evaluated for their antibacterial activity. The structures of the synthesized chalcones were established by IR and ^1^H-NMR analysis. The biological data shows that compounds **p_5_**, **f_6_** and **t_5_** had strong activities against both susceptible and resistant *Staphylococcus aureus *strains, but not activity against a vancomycin and methicillin resistant *Staphylococcus aureus* isolated from a human sample. The structure and activity relationships confirmed that compounds **f_5_**, **f_6_** and **t_5_** are potential candidates for future drug discovery and development.

## 1. Introduction

In the past, many decades since penicillin was discovered and introduced as a powerful antibacterial agent, antibiotics have become critical in the fight against infectious diseases caused by bacteria and other microbes. However, widespread antibiotic use has promoted the emergence of antibiotic-resistant pathogens, including multidrug resistant strains [1,2,3]. At present, the appearance of more and more pathogenetic bacterial species resistant to conventional antibiotics has resulted in either high expenses or failure in the treatment of infectious diseases. An alarming increase in resistance of bacteria that cause community acquired infections has also been documented, especially in Staphylococci and Pneumococci, which are prevalent causes of disease and mortality. In addition, the risk of opportunistic fungal infections increases rapidly accompanied with AIDS disease, and as an obviously consequence invasive infections represent a major cause of mortality for these patients [4]. With the emergence of new microbial strains resistant to many conventional available antibiotics there is growing interest in the discovery of new antibacterial agents [1,2]. According to the known structure and activity relationships, it is considered that certain small heterocyclic molecules act as highly functionalized scaffolds and are known pharmacophores of a number of biologically active and medicinally useful molecules [5,6].

**Figure 1:**
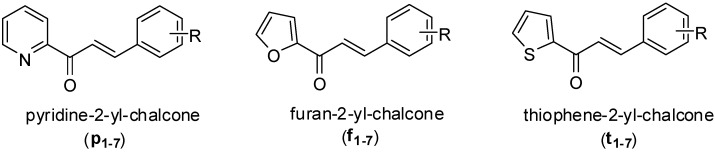
Structure of heterocyclic chalcones obtained from the Claisen Schmidt condensation between heteroaryl methyl ketones and substituted benzaldehydes.

Chalcones (1,3-diphenyl-propene-1-one) belonging to the flavonoid family, are natural and synthetic products that have been reviewed for their wide range of biological activities as antibacterial [7,8], anti-tumor [9,10,11], anti-inflammatory [12,13,14] and antioxidant agents [15,16,17,18], *etc*. Although studies on the bioavailability of heterocyclic chalcones from natural sources are limited, they have been reported as having a wide range of biological activities, especially antibacterial [19,20,21,22,23,24,25,26,27,28], and antifungal activities [29]. In an effort to diversify the biological activities of conventional chalcones, a series of heterocyclic chalcone analogues in which an electron rich nitrogen or oxygen as well as thiophene heterocycle replaces the benzene ring were synthesized. Herein, we report the synthesis of some novel heterocyclic chalcone analogues (Figure 1) using a conventional base catalyzed Claisen Schmidt condensation reaction and their possible antibacterial activity alone and in combination with antibiotics.

## 2. Results and Discussion

### 2.1. Chemistry

**Scheme 1:**
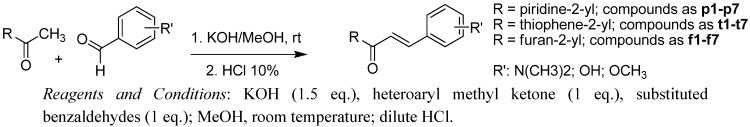
Claisen-Schmidt condensation.

A number of known and novel heterocyclic chalcone analogues were prepared via Claisen Schmidt condensation reactions [14,30] between appropriate benzaldehydes and heteroaryl methyl ketones like pyridine-2-yl methyl ketone, thiophene-2-yl methyl ketone and furan-2-yl methyl ketone (Scheme 1). The purity of the products and progress of the reaction was checked by TLC on silica gel plates. The condensation step was carried out over 6 h to 36 h. After recrystallization from methanol all corresponding chalcones were obtained in 43–63% yields.

The structures of the synthesized compounds were confirmed by IR, ^1^H-NMR and mass spectrometry measurements. The ^1^H-NMR spectrum of the synthesized chalcones displayed two doublets at δ 7.2–8.4 ppm with a characteristic coupling constant (*J*) of 15–16 Hz, which confirms the formation of chalcones (possessing a α,β-unsaturated ketone). This higher coupling constant value indicates all synthetic compounds were geometrically pure and were exclusively *trans* (*E*) isomers. This condensation produced three series of heterocyclic chalcones, which differ in the A ring like pyridine-2-yl-chalcones (**p_1-7_**), furan-2-yl-chalcones (**f_1-7_**) and thiophene-2-yl-chalcone (**t_1-7_**), as listed in Table 1.

### 2.2. Antimicrobial Activity

The antimicrobial assays were carried out by the dilution method using the following bacterial strains: methicillin-sensitive *Staphylococcus aureus* ATCC 29213 (SA or MSSA), methicillin-resistant *Staphylococcus aureus* ATCC 43300 (MRSA) and a vancomycin and methicillin resistant *Staphylococcus aureus* isolated from a human sample. Table 2 summarizes the results obtained for the MICs of the heterocyclic chalcones against the three bacterial strains. Of the 21 heterocyclic chalcones, five compounds had strong activity against the tested *S. aureus*, including two compounds pertaining to the pyridine-2-yl-chalcone group (**p_5_** and **p_6_**), two compounds pertaining to the furan-2-yl-chalcone group (**f_5_**, **f_6_**) and one compound pertaining to the thiophene-2-yl- chalcone group (**t_5_**).

Results of determining the antibacterial coordination ability of the test substances and antibiotics are shown in Table 3. Interestingly, while the five abovementioned chalcones (**p_5_**, **p_6_**, **f_5_**, **f_6_**, **t_5_**) showed moderate antibacterial effects, they might increase dramatically the activity of some combined antibiotics. The chalcones in these combinations also exhibited an antibacterial effect better than that seen when they were used alone (Table 4). In the checkerboard technique, results of quantitatively determining antibacterial coordination ability proved that combinations between antibiotics and chalcone derivatives **f_5_** and **p_6_** had synergistic effects against MRSA ATCC 43300. Such combinations are mixtures of compound **f_5_** and oxacillin; compound **p_6_** and vancomycin. However, no synergistic effect of the combinations of the synthesized heterocylic chalcones and test antibiotics could be found on the local hospital isolated MRSA.

**Table 1:** The structures of the 21 chalcones with their substitution patterns. 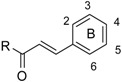

Compounds	A ring (R)	Substitution in B ring				Formula	Isolated yield (%)
		C2	C3	C4	C5		
**p1**	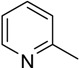	H	NO_2_	H	H	C_14_H_10_N_2_O_3_	45
**p2**		H	H	N(CH_3_)_2_	H	C_16_H_16_N_2_O	62
**p3**		H	H	OCH_3_	OCH_3_	C_16_H_15_NO_3_	58
**p4**		H	OCH_3_	OCH_3_	OCH_3_	C_17_H_17_NO_4_	51
**p5**		OH	H	H	H	C_14_H_11_NO_2_	51
**p6**		H	OH	H	H	C_14_H_11_NO_2_	50
**p7**		H	H	OH	H	C_14_H_11_NO_2_	58
**f1**	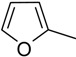	H	NO_2_	H	H	C_13_H_9_NO_4_	43
**f2**		H	H	N(CH_3_)_2_	H	C_15_H_15_O_2_	61
**f3**		H	H	OCH_3_	OCH_3_	C_15_H_14_O_4_	55
**f4**		H	OCH_3_	OCH_3_	OCH_3_	C_16_H_16_O_3_	51
**f5**		OH	H	H	H	C_13_H_10_O_5_	54
**f6**		H	OH	H	H	C_13_H_10_O_3_	58
**f7**		H	H	OH	H	C_13_H_10_O_3_	62
**t1**	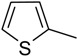	H	NO_2_	H	H	C_13_H_9_NO_3_S	46
**t2**		H	H	N(CH_3_)_2_	H	C_15_H_15_NOS	63
**t3**		H	H	OCH_3_	OCH_3_	C_15_H_14_O_3_S	50
**t4**		H	OCH_3_	OCH_3_	OCH_3_	C_16_H_16_O_4_S	53
**t5**		OH	H	H	H	C_13_H_10_O_2_S	55
**t6**		H	OH	H	H	C_13_H_10_O_2_S	52
**t7**		H	H	OH	H	C_13_H_10_O_2_S	58

**Table 2:** Anti-*Staphylococcus aureus* activity of the synthetic compounds (minimum inhibitory concentration in µg/mL).

Chalcones ^a^\Bacteria	Pyridin-2yl-chalcones			Furan-2yl-chalcones			Thiophen-2yl-chalcones	
	p_5_	p_6_	p_6_	f_5_	f_6_	f_7_	t_5_	t_6_
MSSA ATCC 25923	128	64	512	64	128	256	32	256
MRSA ATCC 44330	128	128	512	64	128	256	64	128
MRSA-I (isolated) ^b^	-	64	-	32	512	-	256	-

^a^: the remaining compounds (**p_1-4_; f_1-4_; t_1-4_** and **t_7_**) were inactive at a concentration of 512 µg/mL; ^b^: isolated MRSA (MICs: oxacillin 256; vancomycin 64 µg/mL); (-): inactive at a concentration of 512 µg/mL; ND: not determined.

**Table 3:** Antibacterial coordination ability results.

Antibiotics	p_6_			f_5_			f_6_			t_5_		
	SA	MRSA	Isolated MRSA	SA	MRSA	Isolated MRSA	SA	MRSA	Isolated MRSA	SA	MRSA	Isolated MRSA
Vancomycin	-	+	-	-	+	-	-	+	-	+	+	-
Doxycycline	-	-	-	-	-	-	-	-	-	-	-	-
Ciprofloxacin	+	-	-	-	-	-	+	-	-	-	-	-
Gentamicin	-	-	-	-	-	-	-	-	-	-	-	-
Chloramphenicol	-	-	-	-	-	-	-	-	-	-	-	-
Oxacicllin	-	-	-	-	+	-	-	+	-	-	+	-
Erythromycin	-	-	-	-	-	-	-	-	-	+	-	-

(SA): MSSA ATCC 25923; (MRSA): MRSA ATCC 43300; (isolated MRSA): MRSA with MICs of oxacicllin 256; 64 µg/mL and vancomycin 64 µg/mL.

**Table 4:** Anti-SA and anti-MRSA activity of chalcones in combination with antibiotics.

Mix ^a^	MSSA ATCC 25923					MRSA ATCC 43300				
	MICs (µg/mL)		FICI	Interpretation	Increasing rate (fold) ^b^	MICs (µg/mL)		FICI	Interpretation	Increasing rate (fold) ^b^
	Alone	Mix ^a^				Alone	Mix ^a^			
**p_6_**	64	32	0.75	Additive		ND	ND	ND	ND	ND
Cipro	0.5	0.125								
**p_6_**	ND	ND	ND	ND	ND	128	32	0.31	Synergistic	4
Vanco						1	0.0625			16
**f_5_**	ND	ND	ND	ND	ND	64	16	0.31	Synergistic	4
Oxa						2	0.125			16
**f_6_**	128	64	0.75	Additive	2	ND	ND	ND	ND	ND
Cipro	0.5	0.125			4					
**f_6_**	ND	ND	ND	ND	ND	128	64	0.625	Additive	2
Vanco						1	0.125			8
**f_6_**	ND	ND	ND	ND	ND	128	64	0.56	Additive	2
Oxa						2	0.125			16
**t_5_**	32	32	1.06	Indifferent	1	ND	ND	ND	ND	ND
Ery	0.5	0.03			1.67					
**t_5_**	32	16	1	Additive	2	ND	ND	ND	ND	ND
Cipro	0.5	0.25			2					
**t_5_**	ND	ND	ND	ND	ND	64	32	1.0	Additive	2
Vanco						1	0.5			2
**t_5_**	ND	ND	ND	ND	ND	64	32	0.56	Additive	2
Oxa						2	0.125			16

^(a)^ Mix = Combination; ^(b)^ Rate in increasing antibacterial activity of antibiotic/chalcone in combination compared to that of in alone (fold); ND = not dertemined. Vanco: vancomycin; Cipro: ciprofloxacin; Ery: erythromycin; Oxa: oxacillin.

Some synthesized heterocyclic chalcones exhibit strong antibacterial activities and may act by damaging the cell wall of SA, which is clearly similar to the observed mechanism of a well-known cell membrane permeabilizer, polymyxin B [27]. However, the molecular mechanism of action is not yet clearly understood. Some preliminary remarks on structure-activity relationship can be drawn from the bioactivity results as follows:

(i) A free hydroxyl group in position 2/3 in the B ring (phenyl moiety) appears to be very important for anti-*Staphylococcus aureus* activity (**p_5_**, **p_6_**, **f_5_**, **f_6_**, **t_5_**). In case of substitution of a hydroxyl group in position 4 (**p_7_**, **f_7_**, **t_7_**), chalcones were inactive on the tested *Staphylococcus aureus* strains.
(ii) Substitution in the B ring with a nitro group in position 3 (as in **p_1_**, **f_1_**, **t_1_**) or with two/three methoxyl groups in positions 3/4/5 (as in **p_3_**, **p_4_**, **f_3_**, **f_4_**, **t_3_** and **t_4_**) might also be responsible for the decrease in the anti-*Staphylococcus aureus* activity;
(iii) The chalcones possessing two/three methoxy groups in B ring were inactive, regardless of their A ring structures. This means that methoxy groups seem to abolish the hydrophilic property of the phenol hydroxy moiety which can affect penetration of antibiotics through bacterial cell walls.
(iv) The chalcones possessing a hydroxyl group in B ring (**p_6_**, **f_6_** and **t_5_**) demonstrated strongly positive interactions with antibiotic such as ciprofloxacin on methicillin-sensitive-*Staphylococcus aureus*. These chalcones increased significantly the activity of combined antibiotics like vancomycin and oxacillin up to sixteen-fold on methicillin -resistant-*Staphylococcus aureus*. These combinations could lead to develop new treatments for MRSA infectious diseases;
(v) The analysis of structural influence of A ring on anti-*Staphylococcus aureus* activity suggested that a furan-2-yl moiety may be more important than the thiophene-2-yl or pyridine-2yl- moieties.

## 3. Experimental

### 3.1. General

Melting points (mp) were taken in open capillary tubes using a Gallenkamp apparatus and are uncorrected. The IR spectra were recorded on a Shimadzu FTIR 8201 PC spectrophotometer. ^1^H-NMR spectra were determined on a Bruker Ultrashield 500 spectrometer using tetramethylsilane (TMS) as an internal reference. All the starting materials are commercially available. 

### 3.2. General Procedure for the Synthesis of Chalcones *[14,30]*

A solution of heteroaryl methyl ketone (5 mM) and aromatic aldehyde (5 mM) in methanol (15 mL) was cooled to 5–10 °C in an ice bath. The cooled solution was treated with a small portion of pulverized KOH (10 mM). The reaction mixture was magnetically stirred for 60 min and then left overnight or longer, as monitored by thin layer chromatography using *n*-hexane-acetone (5:1) as developing solvent. The resulting dark solution was diluted with ice water and carefully acidified using dilute hydrochloric acid. The chalcones, which separated as a yellow solid, were collected by filtration after washing with water and further purified by crystallization from methanol. When needed, the crude product was further purified by silica gel column chromatography. 

Characterization data of the synthesized heterocyclic chalcone analogues: 

*1-(Pyridine-2-yl)-3-(3-nitrophenyl)-2-propene-1-one* (**p_1_**). Mp: 180 °C; IR (νcm^−1^, KBr): 1674 (υ_C=O_) and 1616 (υ_C=C_). ^1^H-NMR (DMSO-d_6_, δ ppm): 8.83 (d, *J *= 4.5 Hz, 1H, H_6′_); 8.63 (s, 1H, H_2_); 8.40 (d, *J *= 16 Hz, 1H, H_β_); 8.31 (d, *J *= 7.5 Hz, 1H, H_4_); 8.29 (d, *J *= 2 Hz, 1H, H_3′_); 8.14 (d, *J *= 8 Hz, 1H, H_6_); 8.10 (m, 1H, H_4′_); 7.98 (d, *J *= 16 Hz, 1H, H_α_); 7.77 (t, 1H, H_5_); 7.74 (m, 1H, H_5′_). MS (ESI, positive scan): *m/z* 277.23 [M+Na]^+^.

*1-(Pyridine-2-yl)-3-(4-dimethylaminophenyl)-2-propene-1-one *(**p_2_**). Mp: 138 °C; IR: (νcm^−1^, KBr): 1656 (υ_C=O_), 1604 (υ_C=C_). ^1^H-NMR (DMSO-d_6_, δ ppm): 8.78 (d, *J* = 4.5 Hz, 1H, H_6_); 8.08 (d, *J *= 8 Hz, 1H, H_3′_); 8.02 (d, 1H, H_4′_); 8.01 (d, *J *= 16 Hz, 1H, H_β_); 7.79 (d, *J *= 16 Hz, 1H, H_α_); 7.67-7.66 (m, 1H, H_5’_); 7.65 (d, *J *= 9 Hz, 2H, H_2_ and H_6_); 6.77 (d, *J* = 9 Hz, 2H, H_3_ and H_4_); 3.02 (s, 6H, 2 × CH_3_). MS (ESI, positive scan): *m/z* 255.35 [M+H]^+^.

*1-(Pyridine-2-yl)-3-(3,4-dimethoxyphenyl)-2-propene-1-one* (**p_3_**). Mp: 105 °C; IR (νcm^−1^, KBr): 1666 (υ_C=O_); 1596 (υ_C=C_); 1271 (υ_C-O-_). ^1^H-NMR (DMSO-d_6_, δ ppm): 8.80 (m, 1H, H_6′_); 8.13 (d, *J *= 16 Hz; 1H, H_β_); 8.09 (d, *J *= 7.5 Hz, 1H, H_3′_); 8.04 (m, 1H, H_4′_); 7.82 (d, *J *= 16 Hz, 1H, H_α_); 7.68 (m, 1H, H_5′_); 7.40 (d, *J *= 8 Hz, 1H, H_2_); 7.37 (d, *J *= 1.5 Hz, 1H, H_6_); 7.04 (d, *J *= 8 Hz, 1H, H_5_); 3.83 (s, 6H, 2 × -OCH_3_). MS (ESI, positive scan): *m/z* 292.34 [M+Na]^+^.

*1-(Pyridine-2-yl)-3-(3,4,5-trimethoxyphenyl)-2-propene-1-one *(**p_4_**). Mp: 160 °C; IR (νcm^−1^, KBr): 1668 (υ_C=O_); 1610 (υ_C=C_); 1124 (υ_C-O-_). ^1^H-NMR (CDCl_3_, δ ppm): 8.76 (m, 1H, H_6′_); 8.20 (s, 1H, H_3′_); 8.17 (d, *J *= 16 Hz, 1H, H_β_); 7.89 (t, 1H, H_4′_); 7.87 (d, *J *= 16 Hz, 1H, H_α_); 7.51 (m, 1H, H_5′_); 6.96 (s, 2H, H_2_ and H_6_); 3.94 (s, 6H, 2 × -OCH_3_); 3.91 (s, 3H, -OCH_3_). MS (ESI, positive scan): *m/z* 322.14 [M+H]^+^.

*1-(Pyridine-2-yl)-3-(2-hydroxyphenyl)-2-propene-1-one *(**p_5_**). Mp: 140 °C; IR (νcm^−1^, KBr): 1660 (υ_C=O_); 1591 (υ_C=C_); 3375 (υ_O-H_). ^1^H-NMR (DMSO-d_6_, δppm): 10.32 (s, 1H, -OH); 8.79 (m, 1H, H_6′_); 8.30 (d, *J *= 16 Hz, 1H, H_β_); 8.10 (d, *J *= 1 Hz, 6.5 Hz, 1H, H_3′_); 8.08 (m, 1H, H_4′_); 8.04 (m, 1H, H_5′_); 7.70 (d, *J *= 1 Hz, 7.5 Hz, 1H, H_3_); 7.67 (d, *J *= 16 Hz, 1H, H_α_); 7.29 (m, 1H, H_4_); 6.96 (d, *J *= 1 Hz, 8 Hz, 1H, H_6_); 6.90 (t, 1H, H_5_). MS (ESI, positive scan): *m/z* 248.07 [M+Na]^+^.

*1-(Pyridine-2-yl)-3-(3-hydroxyphenyl)-2-propen-1-one* (**p_6_**). Mp: 131 °C; IR (νcm^−1^, KBr): 1660 (υ_C=O_); 1566 (υ_C=C_); 3359 (υ_O-H_). ^1^H-NMR (DMSO-d_6_, δ ppm): 9.70 (s, 1H, OH); 8.80 (d, *J *= 4.5 Hz, 1H, H_6′_); 8.19 (d, *J *= 16.5 Hz, 1H, H_β_); 8.10 (d, *J *= 8 Hz, 1H, H_3′_); 8.05 (t, 1H, H_4′_); 7.75 (d, *J *= 16 Hz, 1H, H_α_); 7.69 (m, 1H, H_5′_); 7.27 (t, 1H, H_5_); 7.23 (d, *J *= 8 Hz, 1H, H_6_); 7.18 (s, 1H, H_2_); 6.88 (d, *J* = 1 Hz, 7.5 Hz, 1H, H_4_). MS (ESI, positive scan): *m/z* 248.07 [M+Na]^+^.

*1-(pyridine-2-yl)-3-(4-hydroxyphenyl)-2-propene-1-one* (**p_7_**). Mp: 137 °C; IR (νcm^−1^, KBr): 1664 (υ_C=O_); 1579 (υ_C=C_); 3100 (υ_O-H_). ^1^H-NMR (DMSO-d_6_, δ ppm): 10.12 (s, 1H, -OH); 8.77 (d, *J *= 5.5 Hz, 1H, H_6′_); 8.08 (d, *J *= 16 Hz, 1H, H_β_); 8.03 (m, 2H, H_3′_, H_4′_); 7.77 (d, *J *= 16 Hz, 1H, H_α_); 7.71 (m, 1H, H_5′_); 7.69 (d, 2H, H_2_, H_6_); 6.84 (d, 2H, H_3_, H_5_). MS (ESI, positive scan): *m/z* 248.07 [M+Na]^+^.

*1-(Furan-2-yl)-3-(3-nitrophenyl)-2-propene-1-one *(**f_1_**). Mp: 185 °C; IR (νcm^−1^, KBr): 1656 (υ_C=O_); 1604 (υ_C=C_). ^1^H-NMR (DMSO-d_6_, δ ppm): 8.73 (s, 1H, H_5’_); 8.31 (d, *J *= 8 Hz, 1H, H_4_); 8.27 (d, *J *= 2 Hz, 8 Hz, 1H, H_6_); 8.10 (t, 1H, H_2_); 7.94 (d, *J *= 3.5 Hz, 1H, H_3′_); 7.91 (d, *J *= 16 Hz, 1H, H_β_); 7.85 (d, *J *= 16 Hz, 1H, H_α_); 7.76 (t, 1H, H_5_); 6.82 (m, 1H, H_4′_). MS (ESI, positive scan): *m/z* 266.2 [M+Na]^+^.

*1-(Furan-2-yl)-3-(4-dimethylaminophenyl)-2-propene-1-one* (**f_2_**). Mp: 104 °C; IR (νcm^−1^, KBr): 1643 (υ_C=O_); 1612 (υ_C=C_). ^1^H-NMR (DMSO-*_d6_*, δ ppm): 8.00 (d, *J *= 1 Hz, 1H, H_5’_); 7.67 (d, *J *= 16 Hz, 1H, H_β_); 7.66 (d, *J *= 9 Hz, 3H, H_2_, H_6_ and H_3′_); 7.40 (d, *J *= 16 Hz, 1H, H_α_); 6.76 (m, 3H, H_3_, H_5_ and H_4′_); 3.00 (s, 6H, 2 × -CH_3_). MS (ESI, positive scan): *m/z* 242.2 [M+H]^+^.

*1-(Furan-2-yl)-3-(3,4-dimethoxyphenyl)-2-propene-1-one* (**f_3_**). Mp: 105 °C; IR (νcm^−1^, KBr): 1651 (υ_C=O_); 1595 (υ_C=C_); 1263(υ_C-O-_). ^1^H-NMR (DMSO-d_6_, δ ppm): 8.04 (d, *J *= 1 Hz, 1H, H_5′_); 7.77 (d, *J *= 3 Hz; 1H, H_3′_); 7.70 (d, *J *= 16 Hz, 1H, H_β_); 7.56 (d, *J *= 16 Hz, 1H, H_α_); 7.47 (s, 1H, H_2_); 7.37 (m, 1H, H_6_); 7.02 (d, *J *= 8.5 Hz, 1H, H_5_); 6.78 (m, 1H, H_4′_); 3.83 (s, 6H, 2 × -OCH_3_). MS (ESI, positive scan): *m/z* 281.4 [M+Na]^+^.

*1-(Furan-2-yl)-3-(3,4,5-trimethoxyphenyl)-2-propene-1-one *(**f_4_**). Mp: 158 °C; IR (νcm^−1^, KBr): 1654 (υ_C=O_); 1604 (υ_C=C_); 1122 (υ_C-O-_). ^1^H-NMR (CDCl_3_, δ ppm): 7.80 (d, *J *= 16 Hz, 1H, H_β_); 7.66 (d, *J *= 1 Hz, 1H, H_5′_); 7.34 (d, *J *= 16 Hz, 1H, H_α_); 7.34 (d, *J *=3.5 Hz, 1H, H_3′_); 6.88 (s, 2H, H_2_ and H_6_); 6.61 (m, 1H, H_4’_); 3.93 (s, 6H, 2 × -OCH_3_); 3.90 (s, 3H, -OCH_3_). MS (ESI, positive scan): *m/z* 311.06 [M+Na]^+^.

*1-(Furan-2-yl)-3-(2-hydroxyphenyl)-2-propene-1-one *(**f_5_**). Mp: 138 °C; IR (νcm^−1^, KBr): 1643 (υ_C=O_), 1577 (υ_C=C_); 3124 (υ_O-H_). ^1^H-NMR (DMSO-d_6_, δ ppm): 10.24 (s, 1H, -OH); 8.03 (d, *J *= 16 Hz, 1H, H_β_); 8.03 (d, *J *= 1 Hz, 7.5 Hz, 1H, H_5′_); 7.78 (d, *J *= 1.5 Hz, 6 Hz, 1H, H_6′_); 7.67 (d, *J *= 3.5 Hz, 1H, H_3′_); 7.64 (d, *J *= 16 Hz, 1H, H_α_); 7.27 (m, 1H, H_4_); 6.93 (d, *J *= 0.5 Hz, 8Hz, 1H, H_3_); 6.87 (t, 1H, H_4′_); 6.76 (m, 1H, H_5_). MS (ESI, positive scan): *m/z* 248.07 [M+Na]^+^.

*1-(Furan-2-yl)-3-(3-hydroxyphenyl)-2-propene-1-one *(**f_6_**). Mp: 141 °C; IR (νcm^−1^, KBr): 1643 (υ_C=O_); 1577 (υ_C=C_); 3309 (υ_O-H_). ^1^H-NMR (DMSO-d_6_, δ ppm): 9.61 (s, 1H, -OH); 8.05 (d, 1H, H_5′_); 7.79 (d, 1H, H_3′_); 7.65 (d, *J *= 16 Hz, 1H, H_β_); 7.58 (d, *J *= 16 Hz, 1H, H_α_); 7.25 (t, 1H, H_4′_); 7.25 (d, 1H, H_6_); 7.19 (s, 1H, H_2_); 6.87 (m, 1H, H_5_); 6.78 (m, 1H, H_4_). MS (ESI, positive scan): *m/z* 248.07 [M+Na]^+^.

*1-(Furan-2-yl)-3-(4-hydroxyphenyl)-2-propene-1-one *(**f_7_**). Mp: 142 °C; IR (νcm^−1^, KBr): 1647 (υ_C=O_); 1610 (υ_C=C_); 3200 (υ_O-H_). ^1^H-NMR (DMSO-d_6_, δ ppm): 10.09 (s, 1H, -OH); 8.02 (s, 1H, H_5′_); 7.72 (s, 1H, H_3′_); 7.71(d, *J *= 7.5 Hz, 2H, H_2_ and H_6_); 7.66 (d, *J *= 17 Hz, 1H, H_β_); 7.48 (d, *J *= 16.5 Hz, 1H, H_α_); 6.83 (m, 2H, H_3_ and H_5_); 6.76 (m, 1H, H_4′_). MS (ESI, positive scan): *m/z* 248.06 [M+Na]^+^.

*1-(Thiophene-2-yl)-3-(3-nitrophenyl)-2-propene-1-one *(**t_1_**). Mp: 159 °C; IR (νcm^−1^, KBr): 1651 (υ_C=O_); 1600 (υ_C=C_). ^1^H-NMR (DMSO-d_6_, δ ppm): 8.77 (d, *J *= 1.5 Hz, 1H, H_5′_); 8.43 (d, *J *= 5 Hz, 1H, H_3′_); 8.33 (d, *J *= 8 Hz, 1H, H_4_); 8.27 (d, *J *= 2 Hz, 8 Hz, 1H, H_6_); 8.09 (d, *J *= 16 Hz, 1H, H_β_); 8.10 (d, *J* = 1 Hz, 1H, H_2_); 7.85 (d, *J* = 15.5 Hz, 1H, H_α_); 7.77 (t, 1H, H_5_); 7.54 (m, 1H, H_4′_). MS (ESI, positive scan): *m/z* 282.02 [M+Na]^+^.

*1-(Thiophene-2-yl)-3-(4-dimethylaminophenyl)-2-propene-1-one *(**t_2_**). Mp: 131 °C; IR (νcm^−1^, KBr): 1633 (υ_C=O_); 1612 (υ_C=C_). ^1^H-NMR (CDCl_3_, δ ppm): 7.83 (d, *J *= 15.5 Hz, 1H, H*_β_*); 7.83 (d, *J *= 9 Hz, 1H, H_5′_); 7.62 (d, *J* = 6 Hz, 1H, H_3′_); 7.55 (d, *J *= 9 Hz, 2H, H_2_ and H_6_); 7.23 (d, *J *= 15.5 Hz, 1H, H_α_); 7.16 (t, 1H, H_4′_); 6.70 (d, *J *= 8.5 Hz, 2H, H_3_ and H_5_); 3.04 (s, 6H, 2 × -CH_3_). MS (ESI, positive scan): *m/z* 258.08 [M+H]^+^. 

*1-(Thiophene-2-yl)-3-(3,4-dimethoxyphenyl)-2-propene-1-one* (**t_3_**). Mp: 99 °C; IR (νcm^−1^, KBr): 1639 (υ_C=O_); 1573 (υ_C=C_); 1265 (υ_C-O-_). ^1^H-NMR (DMSO-d_6_, δ ppm): 8.32 (m, 1H, H_5′_); 8.03 (m, 1H, H_3′_); 7.75 (d, *J *= 15.5 Hz, 1H, H*_β_*); 7.68 (d, *J *= 15.5 Hz, 1H, H*_α_*); 7.52 (s, 1H, H_2_); 7.40 (m, 1H, H_6_); 7.31 (m, 1H, H_4’_); 7.02 (d, *J *= 8.5 Hz, 1H, H_5_); 3.83 (s, 6H, 2 × -OCH_3_). MS (ESI, positive scan): *m/z* 297.02 [M+Na]^+^.

*1-(Thiophene-2-yl)-3-(3,4,5-trimethoxyphenyl)-2-propene-1-one *(**t_4_**). Mp: 156 °C; IR (νcm^−1^, KBr): 1645 (υ_C=O_); 1596 (υ_C=C_); 1124 (υ_C-O-_). ^1^H-NMR (CDCl_3_, δ ppm): 7.88 (m, 1H, H_5’_); 7.77 (d, *J* = 15.5 Hz, 1H, H*_β_*); 7.69 (d, *J *= 1 Hz, 5 Hz, 1H, H_3′_); 7.30 (d, *J *= 15.5 Hz, 1H, H_α_); 7.19 (t, 1H, H_4′_); 6.87 (s, 2H, H_2_ and H_6_); 3.93 (s, 6H, 2 × -OCH_3_); 3.91 (s, 3H, -OCH_3_). MS (ESI, positive scan): *m/z* 327.04 [M+Na]^+^.

*1-(thiophene-2-yl)-3-(2-hydroxyphenyl)-2-propene-1-one *(**t_5_**). Mp: 158 °C; IR (νcm^−1^, KBr): 1637 (υ_C=O_); 1560 (υ_C=C_); 3332 (υ_O-H_). ^1^H-NMR (DMSO-d_6_, δ ppm): 10.30 (s, 1H, -OH); 8.20 (d, *J *= 1 Hz, 3 Hz, 1H, H_5′_); 8.04 (d, *J *= 16 Hz, 1H, H_β_); 8.03 (d, 1H, H_6_); 7.86 (d, *J *= 1.5 Hz, 8 Hz, 1H, H_3_); 7.77 (d, *J *= 15.5 Hz, 1H, H_α_); 7.26-7.30 (m, 2H, H_4_, H_5_); 6.94 (d, *J *= 1 Hz, 4Hz, H_3_); 6.88 (t, 1H, H_4′_). MS (ESI, positive scan): *m/z* 253. [M+Na]^+^.

*1-(Thiophene-2-yl)-3-(3-hydroxyphenyl)-2-propene-1-one* (**t_6_**). Mp: 130 °C; IR (νcm^−1^, KBr): 1643 (υ_C=O_); 1577 (υ_C=C_); 3334 (υ_O-H_). ^1^H-NMR (DMSO-d_6_, δppm): 9.62 (s, 1H, -OH); 8.31 (d, *J *= 4 Hz, 1H, H_5′_); 8.05 (d, *J *= 5 Hz, 1H, H_3′_); 7.77 (d, *J *= 15.5 Hz, 1H, H_β_); 7.63 (d, *J *= 16 Hz, 1H, H_α_); 7.31 (t, 1H, H_4′_); 7.30 (d, 1H, H_6_); 7.27 (t, 1H, H_5_); 7.23 (s, 1H, H_2_); 6.87 (m, 1H, H_4_). MS (ESI, positive scan): *m/z* 253. [M+Na]^+^.

*1-(Thiophene-2-yl)-3-(4-hydroxyphenyl)-2-propene-1-one *(**t_7_**). Mp: 151 °C; IR (νcm^−1^, KBr): 1643 (υ_C=O_); 1610 (υ_C=C_); 3172 (υ_O-H_). ^1^H-NMR (DMSO-d_6_, δ ppm): 10.09 (s, 1H, -OH); 8.25 (m, 1H, H_5’_); 8.01 (m, 1H, H_3′_); 7.73 (m, 2H, H_2_ and H_6_); 7.65 (s, 2H, H_β_ and H_α_); 6.83 (m, 2H, H_3_ and H_5_). MS (ESI, positive scan): *m/z* 253. [M+Na]^+^.

### 3.3. Antibacterial Activity

#### 3.3.1. Antibiotics and Antibacterial Agents

Standard powder forms of vancomycin and ciprofloxacin (Sigma Chemical Co., Ltd.), chloramphenicol, doxycycline and gentamicin (Shanghai Fine Chemicals Co., Ltd.), were stored at 2 to 8 °C until use. Antibiotics discs like gentamycin (10 µg), cloramphenicol (30 µg), vancomycin (30 µg), clindamycin (2 µg), doxycycline (30 µg), ciprofloxacin (5 µg) and erythromycin (15 µg) were supplied by Nam Khoa Co., Ltd., Vietnam.

Three standard strains of bacteria: *Staphylococcus aureus* ATCC 25923 and methicillin-resistant *Staphylococcus aureus* ATCC 43300 and isolated MDR MRSA preserved and activated atour Department of Microbiology were used in the study. The bacterial culture media were purchased from Merck. Tryptic Soy Agar (TSA) and Tryptic Soy Broth (TSB) were used for bacteria culture and Mueller-Hinton Agar (MHA) was used for testing antibacterial activity.

#### 3.3.2. Antibacterial Susceptibility Testing

The disc diffusion method was carried out for the antibacterial tests [31]. Briefly a suspension of bacterial strains (100 µL) containing 10^6^ cfu/mL of bacteria was spread on Mueller Hinton agar (MHA) medium. The disc (6 mm in diameter), impregnated with 10 µL of the test compound at the concentration of 102.4 mg/mL in DMSO were placed on the inoculated agar. Negative control was prepared using the same solvent (DMSO), which was employed to dissolve the test compounds. Antibiotics (2–30 µg/disc, 6 mm in diameter) were used as references. The inoculated plates were incubated at 35–37 °C for 24 h. The diameter of inhibitory zones (mm) was measured (Table 2).

#### 3.2.3. Qualitative Determination of the Interaction between the Chalcones and Antibiotics

A routine Kirby-Bauer antibiotics susceptibility test [32,33] was performed to determine the inhibitory zone of selected compounds. Briefly, sterile MHA medium is melted and poured into Petri dishes to get an agar layer about 3–4 millimeters thick. A sterile cotton stick is dipped into the prepared bacterial suspension (prepared to a ﬁnal concentration of approximately 10^8^ CFU/mL), then pressed on the tube wall to dry, then the bacteria are spread on the agar surface evenly. Opened boxes are put in the incubator in 3–5 min to drain. Wells (6 mm in diameter) were punched in the agar. The distances from the hole (synthesized chalcone analogues) and disc (antibiotics) were measured so that margins of two antibiotic inhibitory zones would meet or slightly overlap. The chalcone samples prepared in solutions at rational concentration (1,024 µg/mL) in DMSO were filled into the wells. After allowing the test compounds to diffuse into agar (15 min at 37 °C) the plates were further incubated at 37 °C for a period of 18 h (for MSSA) or 24 h (for MRSA). A positive interaction between a specific antibiotic and a test substance was reported when the inhibitory zone of each compound extended towards the other (Table 3).

#### 3.2.4. Measurement of MIC Values

The MICs of antibiotics and selected active chalcones were determined by the microdilution method as described by The National Committee for Clinical Laboratoty Standard (NCCLS) [34,35]. Each test compound was run in duplicate. The test plates were incubated at 35–37 °C for 24 h. The MIC was taken as the minimum concentration of the dilutions that inhibited the growth of the test microorganism. The concentration of the solvents used in the following assays was maintained at less than 2% so that no inhibition of organisms or interference occurred.

#### 3.2.5. Evaluation of Combined Activity

Antibacterial interactions were determined using the checkerboard assay as previously described [36]. The range of tested compounds concentration used in the checkerboard analysis was such that the dilution range encompassed the MIC for each drug used in the analysis. The ﬁrst compound of the combination was serially diluted along the ordinate, while the second compound was diluted along the abscissa. Broth micro-dilution plates were inoculated with each *S. aureus *strain to yield ~5 × 10^5^ CFU/mL in a 100 μL final volume, and incubated for 18 h–24 h at 37 °C. Synergy has been defined as requiring a fourfold reduction in the MIC of both antibiotics in combination, compared with each used alone, measuring the fractional inhibitory concentration index (FICI). The FICI was calculated for each combination (A and B as tested compounds) using the following formula [36]:


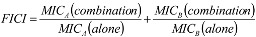


The FICI was interpreted as follows: synergistic, FICI ≤ 0.5; additive, 0.5 < FICI ≤ 1; indifferent, 1 < FICI ≤ 2; antagonistic, FICI > 2.

## 4. Conclusions

The study has proved that the synthesized heterocyclic chalcone analogues have some anti-*Staphyloccus aureus* effects. The results, based on the potentially active chalcone skeleton, have pointed out the importance of the positions of the phenolic hydroxy groups in the B ring for obtaining antibacterial activity. In addition, the furan-2-yl moiety is more active than either the thiophene-2-yl or pyridine-2-yl one in anti-*Staphylococcus aureus* activity. Compounds p_5_, f_6_ and t_5_ exhibited potent inhibitory actvity against methicillin-resistant *Staphylococus aureus* in combination with vancomycin and oxacillin. They may provide a template to design new combinations of antibiotics and non-antibiotics to treatment of *Staphylococus aureus *infections.
